# General Clinico-Pathological Characteristics in Glioblastomas in Correlation with p53 and Ki67

**DOI:** 10.3390/medicina59111918

**Published:** 2023-10-30

**Authors:** Tamás-Csaba Sipos, Attila Kövecsi, Șușu Ovidiu-Ioan, Pap Zsuzsánna

**Affiliations:** 1 Department of Anatomy and Embryology, George Emil Palade University of Medicine, Pharmacy, Sciences and Technology of Târgu Mures, Street Gheorghe Marinescu 38, 540142 Târgu Mures, Romania; tamas.sipos@umfst.ro (T.-C.S.); zsuzsanna.pap@umfst.ro (P.Z.); 2 Department of Pathology, George Emil Palade University of Medicine, Pharmacy, Sciences and Technology of Târgu Mures, Street Gheorghe Marinescu 38, 540142 Târgu Mures, Romania; attila.kovecsi@umfst.ro

**Keywords:** IDH-1, ATRX, glioblastoma, p53, Ki67

## Abstract

*Introduction:* A glioblastoma is an intra-axial brain tumour of glial origin that belongs to the category of diffuse gliomas and is the most common malignant neoplasia of the central nervous system. The rate of survival at 5 years, from the moment of diagnosis, is not higher than 10%. *Materials and methods:* In this retrospective study, fifty-four patients diagnosed with glioblastoma, from the Pathology Department of the County Emergency Clinical Hospital of Târgu Mureș, between 2014 and 2017 were included. We studied the clinico-pathological data (age, gender, location, and laterality) and, respectively, the immunoexpression of p53, Ki67, ATRX, and IDH-1 proteins. *Results:* We observed a statistically significant association between the laterality of the tumour according to the age groups, with the localization on the right side being more frequent in the age group below 65 years of age, while the involvement of the left hemisphere was more prevalent in those over 65 years. Out of the total 54 cases, 87.04% were found to be primary glioblastomas; more than 70% of the cases were ATRX immunopositive; almost 80% of the glioblastomas studied had wild-type p53 profile; and 35% of the cases were found to have a Ki67 index greater than 20%. A statistically significant association between gender and ATRX mutation was found; female cases were ATRX immunopositive in 92% of the cases. Almost 70% of the cases were both IDH-1 and p53 wild-type, and we observed the presence of both mutations in only 3.7% of the cases. Approximately 83% of primary glioblastomas were ATRX positive, respectively, and all IDH-1 mutant cases were ATRX negative. *Conclusions:* Glioblastomas still represent a multidisciplinary challenge considering their reserved prognosis. In this study, we described the most common clinico-pathological characteristics and IHC marker expression profiles, highlighting a variety of percentage ranges in primary and secondary glioblastomas. Given the small number of studied cases, further prospective studies on larger cohorts are needed in the future to evaluate the role of these immunohistochemical markers as prognostic factors for survival or recurrence.

## 1. Introduction

Glioblastoma is characterized as an intra-axial brain tumour of glial origin, which is a subtype category of diffuse gliomas [1], which are represented as a proportion of 48.6% of all malignant tumours of the central nervous system [2]. As the most common intracranial tumour, its incidence increases steadily with advancing age. Glioblastomas can appear at any time during the lifespan, with younger age being associated with a more favourable prognosis. At the time of diagnosis, most patients are over 65 years old. The sex ratio is 1.7 in favour of men [3]; this tumour is almost twice as common in Caucasians than in Afro-American populations [2]. The incidence of glioblastomas in the US population is 3.23/100,000 inhabitants; it is more frequent in the white population [2].

Sometimes glioblastomas can be multifocal or multicentric, but most of the time they are represented by a solitary diffuse lesion [4]. Multifocal glioblastomas show genetic alterations similar to unifocal primary glioblastomas. In contrast, a different genetic profile has been described in multicentric glioblastomas [5]. Regarding the heterogeneity of the various genetic alterations that can occur in glioblastomas, with prognostic or therapeutic relevance, they are represented, among others, by the isocitrate dehydrogenase mutation (IDH-1) or the p53 mutation [6].

Depending on the presence or the absence of IDH-1 or IDH-2 gene mutations, IDH-1 wild-type glioblastomas can be distinguished, representing more than 90% of the cases; additionally, glioblastomas with IDH-1 gene mutations are more frequent in young patients. The survival rate at 5 years, from the moment of diagnosis, is not higher than 10% [7,8,9]. The presence of the IDH-1 mutation confers a longer survival period (31 months) compared to IDH-1 wild-type glioblastomas, where the survival rate does not exceed 15 months [6]. Numerous studies are ongoing regarding treatment opportunities with IDH-1 inhibitors, but their efficacy is considered to be controversial [10].

P53 is a tumour suppressor also called the “guardian of the genome” that regulates several genes involved in the cell cycle or apoptosis, with a role in suppressing tumourigenesis. P53 mutation confers an advantage in tumour cell proliferation and facilitates the malignant transformation of primary cortical astrocytes. The presence of point mutations of the p53 gene is more commonly associated with IDH-1 mutation (65–90%), as opposed to primary glioblastomas (30%) [10,11,12]. Often IDH-1 and p53 mutations are also associated with the presence of ATRX mutation [13]. ATRX mutation occurs in approximately 57% of secondary glioblastoma cases, being more frequent in IDH-1 wild-type glioblastomas. The presence of the ATRX mutation may determine a more favourable prognosis [14].

Ki-67 is a nuclear protein associated with the proliferation phase of a physiological cell cycle, which is specific to the normal or the tumour cell expressing this protein [15,16]. The Ki-67 proliferative index expressed by tumour cells varies between 15% and 40% in most glioblastomas. Some authors have stated that IDH-1 wild-type glioblastomas show a high expression of Ki-67. Others have suggested an association between the tumour size and the increased Ki-67 index in the case of primary glioblastomas, which is consequently associated with an increased risk of recurrence and a less favourable prognosis [10].

Other studies, on the other hand, have demonstrated that the value of the Ki67 index has no role in determining patient prognosis. A high Ki67 index can correlate with a favourable prognosis but also with an unfavourable one, considering that glioblastomas can be resistant or sensitive to adjuvant therapy [10].

In the case of glioblastomas, a multidisciplinary treatment plan is applied consisting of several stages. Initially, if the tumour is operable, the optimal treatment strategy is represented by surgical intervention. Later, the patient follows oncological therapy consisting of radiotherapy and adjuvant chemotherapy. Temozolomide (TMZ) is a cytostatic agent approved for the treatment of glioblastomas, which causes alterations at the level of DNA, that stimulate cell apoptosis. The methylation status of the MGMT promoter of tumour cells can determine resistance to TMZ treatment, favouring the reduction of the therapeutic effect associated with relapse, but the results described in the medical literature often show discordance in this context [17,18,19].

The aim of this study was to determine the main clinico-pathological and immunohistochemical characteristics (IDH-1, ATRX, p53, and Ki-67) of glioblastomas and compare the obtained results with data from the medical literature. To evaluate the most common tumour immunophenotypes, we studied the immunoexpression of some markers frequently used in the histopathological diagnosis of glioblastomas: IDH-1, ATRX, p53, and Ki-67.

## 2. Materials and Methods

### 2.1. Clinical Data

In this retrospective study, during the period 2014–2017, a group of 54 patients diagnosed with glioblastoma was selected within the Pathology Department of the County Emergency Clinical Hospital of Târgu Mureș. The inclusion criteria were as follows: (1) histopathological confirmation of glioblastoma without any previous diagnosis or treatment of a CNS tumour, (2) no history of brain biopsy, (3) tumour tissue available in at least two paraffin blocks for determination of the immunoexpression of IDH1-R132, ATRX, Ki67, and p53. Histopathological diagnoses were re-evaluated by a neuropathologist according to the classification of nervous system tumours developed by the World Health Organization.

### 2.2. Immunohistochemistry

The intra-operative samples were fixed in formalin, embedded in paraffin, and later sectioned at a thickness of 3μm. The sections obtained followed the standard procedure of deparaffinization and rehydration. Endogenous peroxidase was blocked by applying a 10 min bath treatment of 3% H_2_O_2_. Antigen retrieval was performed by steaming under pressure for 25 min in citrate solution (pH 6). We used IDH1R132H mouse monoclonal antibody, IHC132 (BioSB) clone, 1:25 dilution, hPh, 60 min; ATRX mouse monoclonal antibody, BSB-108 (BioSB) clone, 1:50 dilution, hPh, 60 min; Ki67 mouse monoclonal antibody, MM1 (Novocastra, Leica Biosystems) clone, 1:150 dilution, hPh, 60 min; and p53 mouse monoclonal antibody, DO7 (BioSB) clone, 1:800 dilution, hPh, 60 min. The EnVision Flex/horseradish peroxidase (HRP) secondary system, (Dako, 30 min) was used for signal amplification, and the chromogen 3,3′-diaminobenzidine (DAB) was used for primary antibody detection. Later, the slides were stained with hematoxylin.

### 2.3. Slide Examination

A preliminary examination of the slides was performed using an Olympus BX61 microscope. Immunohistochemical reactions regarding p53 and Ki67 were evaluated and counted individually under the supervision of an experienced neuropathologist, the Ki-67 proliferation index was determined as the percentage of positively stained tumour cells (regardless of intensity) per 1000 cells. The presence of p53 was determined using the percentage of cells showing the immunolabel relative to 200 cells in 5 fields. We considered the immunoexpression to be negative if immunolabeling was <10% (wild-type) and positive if it was >10% of cells examined (mutant).

IDH-1 mutation expression was determined by evaluating positively stained tumour cells cytoplasmically and regardless of colour intensity. Cases in which ≥10% of cells were stained were defined as positive (IDH-1 mutant); cases where the value did not exceed 10% of the tumour cells were considered negative (IDH-1 wild-type).

In the case of the ATRX marker, ATRX gene mutations are followed by the loss of nuclear immunoexpression in tumour cells, while ATRX immunoexpression remains preserved in cells not affected by the tumour, which also represents the positive endogenous control (endothelial cells). Cases with ≥50% stained cells were defined as ATRX positive, and cases with <50% stained cells were defined as ATRX negative (ATRX loss).

### 2.4. Statistical Analysis

Statistical data analysis was performed using GraphPad Prism 9, version 9.4.1 (GraphPad Software Inc, San Diego, CA, USA). A statistical significance was set for a *p* < 0.05 value (95% confidence interval).

### 2.5. Limitation of the Study

Our Study’s Main Limitation is the Small Sample Size.

### 2.6. Ethics Committee

This study was approved by the Ethics Committee of the County Emergency Clinical Hospital of Târgu Mureș.

## 3. Results

### 3.1. The Study of Clinico-pathological Parameters

In our retrospective study, we included 54 cases of glioblastomas, with ages between 8 and 79 years and a slight male predominance of 53.7% (29/54). In total, 66.7% (35/54) of all of the cases were under 65 years of age (Table 1). The majority of female cases were between the ages of 50 and 65, 44% (11/25), the ratio of cases under 50 and over 65 years was equal, 28% (7/25) and 28% (7/25), respectively. In men, the fewest male cases were between 50 and 65 years old, 20.69% (6/29), and most cases were found to be under 50 years old, 41.38% (12/29).

In terms of location, over 40% (22/54) of glioblastomas were found to be located in the temporal lobe, followed by frontal localization (29.6%). The fewest cases were located in the parietal lobe (16.6%) and occipital lobe (13%) (Table 1). In women, the most frequently affected lobe was the temporal lobe in 48% (12/25) of the cases, and in men, it was the frontal lobe in 37.93% (11/29) of the cases. The number of cases in which the tumour was located in the parietal lobe was twice as high in females than in males. In men, the occipital lobe was affected 2.5 times more frequently than in women (Table 2).

Regarding the age range between 50 and 65 years, glioblastomas were found to be more common in the temporal lobe (53%). In patients aged under 50 and over 65, the frontal and temporal lobes were equally affected (under 50 in 36.8% and 36.8%, over 65 in 33.3% and 33.3%). We could not demonstrate a statistically significant correlation between the age and gender of the patients with respect to the location of the tumour (Table 2).

As far as laterality is concerned, both cerebral hemispheres were equally affected. In females the glioblastomas were found in more than 50% (14/25) of the cases in the right cerebral hemisphere, compared to the males where the left cerebral hemisphere was affected in a proportion of 55.17% (16/29). In almost 65% (11/17) of patients aged between 50 and 65 years and in 58% of patients under 50 years the right cerebral hemisphere was affected, the fewest cases in this hemisphere were diagnosed in patients over 65 years old. In contrast, where the left cerebral hemisphere was affected, most cases were over 65 years old (72%), and the fewest were in patients aged between 50 and 65 years (Table 2). Regarding localization in the right cerebral hemisphere, almost 60% of the cases were located in the temporal lobe, whereas glioblastomas in the left hemisphere were more frequent in the frontal, parietal, and occipital lobes. The relationship between the tumour laterality and the age groups was found to be almost statistically significant, with the location on the right side being more frequent in patients under 65 years of age, while the glioblastomas to the left hemisphere was more frequent over 65 years (Table 2).

### 3.2. Immunohistochemical Study

#### 3.2.1. IDH-1 Immunoexpression

IDH-1 mutant glioblastomas were more common in males at a rate of 20.69% (6/29) compared to females where the ratio of these cases was 4% (1/25) (*p* = 0.06). Most cases with IDH-1 mutation were found to be present in patients aged between 50 and 65 years (3/7, 42.8%) (*p* = 0.78). Regarding localization, most cases with primary glioblastomas were located in the temporal lobe (20/47, 42.5%). Most cases presenting the IDH-1 mutation were observed in the frontal lobe, with a proportion of 42.8% (3/7) (*p* = 0.84). More than 53.1% (25/47) of IDH-1 wild-type glioblastomas were located in the right cerebral hemisphere compared to the left hemisphere, where there were 2.5 times more cases of secondary glioblastomas, with a proportion of 71.4% (5/7) (*p* = 0.22) (Table 3).

#### 3.2.2. ATRX Immunoexpression

ATRX immunoexpression was present in more than 70% of glioblastoma cases (39/54), with a higher ratio of positive ATRX cases in those over 65 years of age (35.8%, 14/39) (*p* = 0.8), in females (58.9%, 23/39), and in the case of glioblastomas located in the temporal lobe (43.5%, 17/39) and in the right hemisphere (53.8%, 21/39). The ratio of negative ATRX cases was higher in patients under 50 years of age (40%, 6/15), in males (86.6%, 13/15), and in glioblastomas located in the frontal lobe (46.6%, 7/15) and in the left hemisphere (60%, 9/15) (Table 3). In women, glioblastomas were ATRX positive in over 90% (23/25) of cases; in comparison, in men, ATRX immunoexpression was present in only 55.17% (16/29) of cases. A statistically significant association was observed between gender and ATRX mutation (*p* = 0.026). No statistically significant association was observed between the ATRX mutation and the location and laterality of the tumours (*p* = 0.31 and *p* = 0.36, respectively) (Table 3). In men, positive ATRX cases were more frequent under 50 years and over 65 years, with a proportion of 24.14% (7/29) per age group. In women, positive ATRX cases were more numerous between 50–65 years, with a proportion of 40% (10/25) (Table 4).

ATRX positive and IDH-1 wild-type glioblastomas in women were represented in 92% (23/25) and in men in 55.1% (16/29) of the cases. ATRX negative and IDH-1 mutant glioblastomas were represented in men in 20.6% (6/29) and in women only in 4% (1/25) of the cases (Table 5).

Regarding age, ATRX positive and IDH-1 wild-type glioblastomas were more frequently registered in those over 65 years of age (14/39, 35.8%). IDH-1 mutant and ATRX negative glioblastomas were found to be more common in the age group between 50 and 65 years (3/7, 42.8%) (Table 5).

Regarding primary IDH-1 wild-type glioblastomas, a proportion of 82.98% (39/47) are ATRX immunopositive, and all IDH-1 mutant cases are ATRX negative (*p* = 0.0001) (Table 6).

#### 3.2.3. p53 Immunoexpression

**Figure 1:**
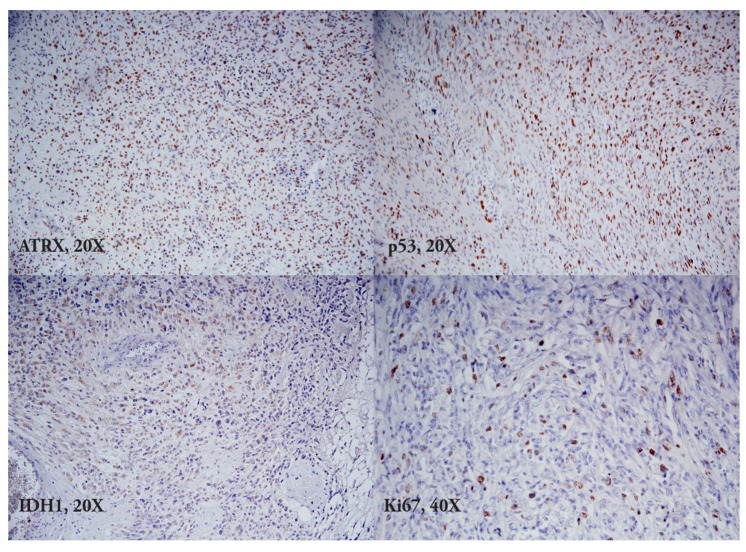
IDH, ATRX, p53 mutant types, and Ki67.

Of the 54 glioblastomas, almost 80% (43/54) had a wild-type profile with regard to the p53 mutation (negative p53 immunoexpression), and 20.3% of the studied cases presented the p53 mutation (11/54) (positive p53 immunoexpression) (Table 3, Figure 1). The p53 mutation was more frequent in patients under the age of 50, 54.5% (6/11), compared to the wild-type p53 glioblastomas, which, in a proportion of 39.5% (17/43), developed in patients aged over 65 years. It was observed that with increasing age, the number of mutant p53 cases decreased (*p* = 0.13). Regarding gender differences, p53 wild-type cases were more frequent in males, 55.8% (24/43), and p53 mutant glioblastomas were slightly more frequent in females (6/11, 54.5%) (Table 3).

As concerns localization, the temporal lobe proved to be the most frequently affected, both in the case of p53 wild-type glioblastomas (17/43, 39.5%) and in the case of p53 mutant (5/11, 45.4%) (*p*= 0.96). The fewest p53 mutant cases were located in the occipital lobe (1/11, 9%). The absence of the p53 mutation showed a slight predominance in the left hemisphere (22/43, 5.11%) and the presence of the mutation in the right hemisphere (6/11, 54.5%) (*p* = 0.73) (Table 3).

Almost 70% (38/54) of the cases were IDH-1 and p53 wild-type, and only 3.7% (2/54) of the glioblastomas were IDH-1 and p53 mutant (Table 6). Among the IDH-1 wild-type glioblastomas, p53 wild-type ones were more common in patients over 65 years of age (15/47, 32%), and p53 mutant ones affected more patients under 50 years of age (5/47, 10.6%). Mutant IDH-1 and p53 glioblastomas developed in patients under 65 years of age (2/7, 28.5%) (Table 7).

The ratio of IDH-1 and p53 wild-type glioblastomas was 69% (20/29) in men and 72% in women (18/25). IDH-1 and p53 mutant glioblastomas represented 6.9% of the cases (2/29) in men and were absent in women (Table 7).

In more than 50% (31/54) out of the total cases examined, there were ATRX positive and p53 wild-type, which were followed in terms of frequency by ATRX negative and p53 wild-type cases (12/54, 22.2%). Only 5.5% (3/54) of glioblastomas were p53 mutants and ATRX negative (Table 6).

Regarding ATRX positive and p53 wild-type cases, they were more common in patients over 65 years of age (13/39, 33%). ATRX negative and p53 mutant glioblastomas affected patients under 65 years of age (3/15, 20%), with ATRX negative p53 mutant cases being absent over 65 years of age. (Table 8)

#### 3.2.4. Ki67 Proliferation Index

In the glioblastoma cases studied, the value of the Ki67 index was <5% in 33.3% (18/54), between 5 and 20% in 31.4% (17/54), and >20% in 35.1% (19/54) of the cases (Table 3). Ki67 values above 20% were more frequent in patients under 50 years of age (8/19, 42.1%), in women (10/19, 52.6%), and in glioblastomas developed in the temporal lobe (9/19, 47.3%) and in the left hemisphere (10/19, 52.6%). Ki67 index values lower than 5% were more frequent in patients over 50 years of age (15/18, 83%), in women (10/18, 55.5%), and in glioblastomas developed in the frontal lobe (6/18, 33.3%) and in the right hemisphere (10/18, 55.5%) (Table 3).

In men, most of the cases without an IDH-1 mutation had a Ki67 index between 5 and 20% (9/29, 31%). Regarding females, IDH-1 wild-type glioblastomas were found in most cases to have a Ki67 index below 5% (10/25, 40%) (Table 9).

ATRX positive cases were more frequent in glioblastomas with a Ki-67 index above 20% (16/39, 41%); comparatively, ATRX negative cases were more frequent in glioblastomas with a Ki67 index below 20% (12/15, 80%) (*p* = 0.34) (Table 6).

ATRX positive glioblastomas with a Ki67 index below 5% and above 20% were more frequent in women than in men (10/25, 40% and 9/25, 36%, respectively). ATRX negative glioblastomas with a Ki67 index below 5% and above 20% were more frequent in men (6/29, 20.6% and 2/29, 6.9%) compared to women (Table 10)

Studying the immunohistochemical results, we could not demonstrate a statistically significant correlation between the Ki-67 index and the presence of IDH-1 (*p* = 0.78) or ATRX (*p* = 0.94) mutations (Table 6).

## 4. Discussion

Glioblastoma (GBM) is the most common primary brain tumour in adults. Glioblastoma as part of the category of diffuse gliomas is associated with an unfavourable prognosis, especially in the case of IDH-1 wild-type glioblastomas, which occurs mainly in patients over 55 years of age, while IDH-1 mutant glioblastomas predominantly appear in younger adults [20]. According to studies in the medical literature, the number of IDH mutant glioblastomas usually does not exceed 10% of all analysed cases [21]. The incidence of mutant IDH-1 glioblastomas reported by Martinez-Lage M et al. and Jakovlevs A et al. did not exceed 5% [22,23]; in the study by Dahlrot et al., it was only 1.65% [15]. In other studies, such as that of Cho et al., this value exceeded 10% (17%) of the studied glioblastomas [24]. According to some studies, patients with IDH-1 wild-type glioblastomas have a lower survival rate compared to mutant IDH-1 glioblastomas [23]. Other studies, on the other hand, state that there is no significant difference in terms of survival between patients regardless of the IDH-1 wild-type and IDH-1 mutant glioblastomas [24].

According to data from the literature, glioblastomas affect patients over 55 years of age [20], the average age being 65 years [2]. There are studies that describe higher or lower values. The mean age reported by Priambada et al. was 45 years and by Qin et al. it was 52 years [25,26]. Higher values were described by Wong et al. (58 years) and by Shies et al. (65 years) [27,28]. Regarding our studied cases, the median age was 55 years. In the study by Haque et al., the majority of glioblastoma patients were aged between 51 and 65 years, which is in contrast with our results, where most cases diagnosed were under 50 years of age [29]. According to Kai et al., among the cases recruited, 47.9% of the subjects were over 65 years old at the time of diagnosis, and Tian et al. described that in 55.2% of the studied glioblastoma cases, patients were aged between 41–60 years [30,31].

Regarding gender distribution, Jakovlevs A observed a slight predominance of females (51.4%), which is in contrast to our study, where the males were more frequently affected by these malignant lesions [23]. Similar to our study, Priambada et al., Qin et al., Wong et al., and Haque et al. have described similar rates regarding glioblastomas that developed in males (≥53%) [25,26,28,29]. In the study by Alimohammadi E et al., in over 60% of the analysed group, glioblastomas predominantly affected the male sex, and almost 10% of them were IDH-1 mutant glioblastomas [32]. Shies et al. state that the survival rate of patients with glioblastomas was not influenced by the age or gender of the patients; in contrast, Wong et al. observed that female patients had a longer survival period compared to males [27,28]. Tian et al. observed that males had a lower survival rate in the age category of 41–60 years and over 60 years compared to female patients in the same age groups [31].

In terms of location, glioblastomas predominantly affect the supratentorial region, but unusual locations have been reported in the cerebellum or brainstem [14]. In most cases, glioblastomas affect only one cerebral lobe, predominantly the frontal or temporal lobe [33]. Qin et al. and Shies et al. observed a more frequent localization in the frontal lobe, followed by the temporal lobe [26,27], which is in contrast to our study, where the most affected lobe was found to be the temporal lobe. In our study, the least affected cerebral lobe was the occipital lobe [26,33]. In contrast to our study, Shies et al. described the fewest cases in the parietal lobe [27]. Alimohammadi et al., similar to our study, reported that the temporal lobe was the most frequently affected, followed by the frontal lobe; they also found that in the case of glioblastomas, there is no significant correlation between tumour location, age, and gender of the patients [32].

Glioblastomas develop in most cases in the form of a solitary diffuse lesion [34]. In some cases, multiple synchronous lesions can be identified, with their incidence being between 2 and 35% [35]. Shies et al. observed that of all glioblastomas studied, 5% have synchronous lesions, compared to the study by Haque et al., where the predominance of these lesions was higher at 17.2% of all cases analysed [27,29]. The survival rate in patients with multicentric glioblastomas is significantly lower than in cases with solitary glioblastomas [36,37]. The meta-analysis by Di Carlo et al. suggests a prevalence of 19% for multifocal and multicentric glioblastomas [38].

In the study of Carlo et al., the median age was 58 years, compared to the group documented by Lasocki et al., where this was almost 65 years [37,38]. In both of the studies, males were predominantly affected, the M:F ratio being 1.26 and 1.32, respectively [37,38]. As for localization, the most frequently affected lobe was the frontal lobe, followed by the temporal lobe, and in terms of laterality, the distribution on both hemispheres was relatively similar. There was no statistically significant association of solitary versus multiple lesions compared with age or lesion location [38]. There were differences between multicentric and multifocal glioblastomas at a molecular level. IDH-1 mutation in newly diagnosed multifocal glioblastomas are uncommon; however, Dono et al. and Lahmi et al. found 3% and 9%, respectively, of multifocal or multicentric glioblastomas to be IDH-1 mutant [39,40].

According to data from the medical literature, genetic and molecular factors have been described to play a role in the diagnosis and prognosis of these lesions and in the effect on the invasive nature of the tumour. These include ATRX or p53 mutation [41]. Current studies have found that there is an association between IDH-1 mutations and ATRX mutations. In rare cases, glioblastomas may show that decreased ATRX expression is associated with a wild-type IDH-1 profile [42]. ATRX loss/ IDH-1 wild-type cases were described in the present study in a proportion of 14.8%. Similar values were also reported in the studies carried out by Ebrahimi A et al., Wiestler B et al., and Pathak P et al. (11%, 10%, and 48%, respectively) [43,44,45].

Kalidindi N et al. reported 80% in terms of IDH-1 and p53 mutant glioblastomas, which is in contrast with our results, where only 28.5% of IDH-1 mutant glioblastomas showed p53 mutation [46].

Considering the fact that the prognosis of patients diagnosed with glioblastomas is unfavourable, two possible outcomes were observed after treatment was applied. Neurosurgical intervention may be followed by tumour progression if adjuvant treatment has not been effective. After adequate oncological treatment, pseudo-progression may occur. It is found in 10–30% of patients with glioblastoma. Currently, there is no biomarker that can distinguish real/actual tumour progression and pseudo-progression. Studies in the medical literature associated p53 overexpression (more than 10% of tumour cells) with the process of pseudo-progression [47]. Montemuro et al. noticed that p53 overexpression was found in cases of relapse, which favoured survival. Another factor involved in the survival rate was the gender of the patients, with women having a longer survival time than men. On the other hand, age or tumour localization did not influence the survival rate [48].

The Ki-67 index is an independent factor in terms of the age and gender of the patients with glioblastomas, and it can provide significant therapeutic and prognostic information [49]. Regarding the Ki67 index, the medical literature states contradictory results. Wong et al. demonstrated that patients with radio- and chemotherapy have a longer survival associated with a high Ki67 index. At the same time, a Ki-67 proliferation index lower than 22% predicts poorer survival in glioblastomas [28]. In contrast, Dalhorth et al. observed that the average Ki67 index value of more than 20% of IDH-1 wild-type glioblastomas predicts a lower survival rate compared to cases where the Ki67 index is lower [15]. Conversely, the metanalysis conducted by Chen et al. suggests, a reserved prognosis regarding the survival of patients with glioblastomas, where the Ki67 index is increased [50].

## 5. Conclusions

Glioblastoma cases have short survival rates from the time of diagnosis. In this retrospective study, we studied the main clinicopathological and immunohistochemical features in 54 cases of glioblastomas. The results obtained regarding age, gender, localization, and laterality are similar to those described in the medical literature, with the observation that glioblastomas involving the left hemisphere develop more frequently over 65 years of age. Most of the studied cases presented IDH-1, ATRX, and p53 wild-type immunophenotype, respectively, at a Ki67 proliferation index above 20%. All IDH-1 mutant cases were ATRX mutant; furthermore, more than 90% of glioblastomas developed in women are ATRX immunopositive.

Given the small number of studied cases, further prospective studies on larger cohorts are needed in the future, to evaluate the role of the studied immunohistochemical markers as prognostic factors for survival or recurrence.

## Figures and Tables

**Table 1:** Clinico-pathological parameters.

Characteristics	Value
**N (primary tumour)**	54
**Median age**	55
**Age classes**	N (%)
<50	19 (35.19)
50–65	17 (31.48)
>65	18 (33.33)
**Gender**	N (%)
Male	29 (53.7)
Female	25 (46.3)
**Male-to-female ratio**	1.16
**Localization (lobe)**	N (%)
Frontal	16 (29.63)
Temporal	22 (40.74)
Parietal	9 (16.67)
Occipital	7 (12.96)
**Laterality**	N (%)
Righ hemisphere	27 (50)
Left hemisphere	27 (50)

**Table 2:** Location and laterality in relation to age and gender.

	AGE (YEARS)			GENDER	
	<50	50–65	>65	Female	Male
**LOCALIZATION**					
TEMPORAL	7	9	6	12	10
FRONTAL	7	3	6	5	11
PARIETAL	3	3	3	6	3
OCCIPITAL	2	2	3	2	5
P (CHI TEST)	**0.86**			**0.21**	
**LATERALITY**					
RIGHT	11	11	5	14	13
LEFT	8	6	13	11	16
P (CHI TEST)	**0.06**			**0.41**	

**Table 3:** IDH-1, ATRX, p53, and Ki67 in correlation with clinico-pathological parameters.

	IDH-1		ATRX		Ki67			p53	
	WILD	MUTANT	POSITIVE	NEGATIVE	<5%	5–20%	>20%	WILD	MUTANT
**LOCALIZATION**									
TEMPORAL	20	2	17	5	5	8	9	17	5
FRONTAL	13	3	9	9	6	6	4	13	3
PARIETAL	8	1	1	8	3	1	5	7	2
OCCIPITAL	6	1	2	5	4	2	1	6	1
**P (CHI TEST)**	0.84		0.31		0.4			0.96	
**AGE (YEARS)**									
<50	17	2	13	6	3	8	8	13	6
50–65	14	3	12	5	8	3	6	13	4
>65	16	2	14	4	7	6	5	17	1
**P (CHI TEST)**	0.78		0.80		0.27			0.13	
**GENDER**									
MALE	23	6	16	13	8	12	9	24	5
FEMALE	24	1	23	2	10	5	10	19	6
**P (CHI TEST)**	0.06		0.026		0.23			0.53	
**LATERALITY**									
RIGHT	25	2	21	6	10	8	9	21	6
LEFT	22	5	18	9	8	9	10	22	5
**P (CHI TEST)**	0.22		0.36		0.84			0.73	

**Table 4:** Relation between age, gender, and ATRX.

	Age	ATRX+	ATRX−
**MALE**	<50	7	5
	50–65	2	4
	>65	7	4
**FEMALE**	<50	6	1
	50–65	10	1
	>65	7	0

**Table 5:** IDH-1 and ATRX in correlation with age and gender.

	IDH-1 WILD TYPE		IDH-1 MUTANT TYPE	
	ATRX+	ATRX−	ATRX+	ATRX−
<50	13	4	0	2
50–65	12	2	0	3
>65	14	2	0	2
**MALE**	16	7	0	6
**FEMALE**	23	1	0	1

**Table 6:** IDH-1, ATRX, p53, and Ki67 correlations.

	IDH-1		ATRX		p53	
	WILD	MUTANT	POSITIVE	NEGATIVE	WILD	MUTANT
**ATRX MUTATION**						
ATRX POSITIVE	39	0				
ATRX NEGATIVE	8	7				
**P (CHI TEST)**	**0.0001**					
**p53**						
p53 MUTANT	9	2	8	3		
p53 WILD TYPE	38	5	31	12		
**P (CHI TEST)**	**0.56**		**0.96**			
**Ki67**						
<5%	16	2	12	6	14	4
5–20%	14	3	11	6	14	3
>20%	17	2	16	3	15	4
**P (CHI TEST)**	**0.78**		**0.34**		**0.94**	

**Table 7:** IDH-1 and p53 in correlation with age and gender.

	IDH-1 WILD TYPE		IDH-1 MUTANT TYPE	
	p53 Wild Type	p53 Mutant Type	p53 Wild Type	P53 Mutant Type
<50	12	5	1	1
50–65	11	3	2	1
>65	15	1	2	0
**MALE**	20	3	4	2
**FEMALE**	18	6	1	0

**Table 8:** Relation between ATRX, p53, and age.

	ATRX+		ATRX−	
	p53 WILD TYPE	p53 MUTANT TYPE	p53 WILD TYPE	p53 MUTANT TYPE
<50	8	5	5	1
50–65	10	2	3	2
>65	13	1	4	0

**Table 9:** Ki67 index in correlation with IDH-1 and gender.

	Ki67	<5%	5–20%	>20%
**MALE**	IDH-1 WILD TYPE	6	9	1
	IDH-1 MUTANT TYPE	2	3	1
**FEMALE**	IDH-1 WILD TYPE	10	5	9
	IDH-1 MUTANT TYPE	0	0	1

**Table 10:** Ki67 index in correlation with ATRX and gender.

	Ki67	<5%	5–20%	>20%
**MALE**	ATRX+	2	7	7
	ATRX−	6	5	2
**FEMALE**	ATRX+	10	4	9
	ATRX−	0	1	1

## Data Availability

All data produced here are available upon request.
